# Social determinants of health are associated with low uptake of daily HIV pre-exposure prophylaxis and future uptake of long-acting formats: a survey of Black and Latina/x/e transfeminine adults in Chicago

**DOI:** 10.3389/fpubh.2025.1626060

**Published:** 2025-09-12

**Authors:** Hale M. Thompson, Tommy Schafer, Reyna Ortiz, J. Silas Leslie, Juan Rivera

**Affiliations:** ^1^Center for Education, Research and Advocacy, Howard Brown Health, Chicago, IL, United States; ^2^Department of Health Behavior, University of Alabama at Birmingham, Birmingham, AL, United States; ^3^TaskForce Prevention and Community Services, Chicago, IL, United States; ^4^Chicago Therapy Collective, Chicago, IL, United States

**Keywords:** transgender (binary and non-binary), HIV prevention (PrEP), social determinants of health, racial inequities, community engagement, implementation science, transfeminine

## Abstract

**Introduction:**

The transgender population in the United States continues to experience disproportionately high HIV prevalence, and Black and Latina transgender women, in particular, account for more than half of the population’s new diagnoses. Uptake of pre-exposure prophylaxis (PrEP), the daily oral HIV prevention pill, among Black and Latina transfeminine adults has lagged. This community-engaged, HIV status-neutral study served as an initial investigation of Black and Latina/x/e transfeminine adults’ attitudes toward various long-acting formats of PrEP relative to the daily pill.

**Methods:**

An online survey was conducted in Chicago, IL, in 2023 (*N* = 198). A multiple logistic regression analysis was conducted to identify factors associationed with the most highly preferred long-acting PrEP format. Second, we compared individuals who would be indicated for daily PrEP based on the 2021 CDC eligibility guidance with those reporting current uptake of the daily pill.

**Results:**

Our findings indicate having a college degree or higher (adjusted odds ratio (aOR) = 6.73 [95% CI: 2.18–20.81], *p* < 0.001) and full-time employment (aOR = 2.70 [95% CI: 1.19–6.17], *p* = 0.018) were associated with reporting a preference for taking the monthly pill while controlling for age group, race, and sexual orientation. Second, those currently taking PrEP were disproportionately stably housed, and 54% reported no CDC-endorsed indications. The positive predictive value of the 2021 CDC PrEP eligibility guidance was 0.30.

**Discussion:**

Similar to other populations vulnerable to HIV, social determinants of health were associated with a preference for the long-acting pill and with current uptake of the daily pill. These findings signal a need for additional research into innovative PrEP implementation strategies that mitigate the structural barriers that transfeminine adults face.

## Introduction

In the United States, transgender women (TW) remain vulnerable to HIV infection (1). According to the Centers for Disease Control (CDC), transgender people constituted 2%, or 671 cases, of all new HIV diagnoses in 2019, while transgender people are estimated to account for 0.6% of the total population (2, 3). Black TW represented 46% of those 671 new diagnoses (3). A longitudinal study (2017–2019) of young TW in San Francisco underscores the CDC findings; in the cohort of 337 18–24-year-old TW, a non-white racial identity, a history of incarceration, and a lack of health insurance were associated with increased HIV incidence (4). Similarly, a recent meta-analysis of 88 studies conducted between 2007 and 2018, estimated laboratory-confirmed HIV prevalence among TW at 14.2% and self-reported prevalence at 21%; the estimates were highest among Black and Latina TW at 44.2 and 25.8%, respectively (1).

To address these ongoing HIV disparities, in 2019, the federal government announced a national strategic plan, known as “Ending the HIV Epidemic” (EHE). To reduce new HIV infections by 90% by 2030, EHE’s prevention pillar aims to expand access to and uptake of antiretroviral HIV pre-exposure prophylaxis, known as PrEP (5). Determined efficacious as an oral pill taken daily (6–8), PrEP has played a critically effective role in primary HIV prevention among white gay men in the U.S. since it became widely available in 2014 (9, 10). PrEP has been less effective in real-world settings with other priority populations, such as Black and Latina transgender women, due to implementation challenges with uptake, adherence, and retention in care (5, 11, 12). Poverty and structural racism, which have shaped access to healthcare, have also driven disparities in PrEP uptake (12, 13). Notably, HIV testing, uptake of PrEP, and concentrations of PrEP medication remain lower among Black and Latina TW relative to other priority populations (11, 14–20).

Long-acting PrEP formulations represent a novel HIV prevention medication that may reduce new infections among Black and Latina transfeminine populations. Transfeminine is defined here as inclusive of multiple gender identities—for example, TW, non-binary, gender non-conforming, and woman—among individuals assigned male at birth but who do not identify as men. As of 2022, only a bi-monthly injectable format, i.e., cabotegravir, had been demonstrated effective with and available for TW (21), although a semi-annual injectable format was approved in June 2025 (22). Many other formats, such as the monthly oral pill and the transdermal patch, remain in the research pipeline. Some studies have highlighted transgender participants’ favorable perceptions of long-acting formats (23–27). At the same time, these studies and others have found that, such as the daily oral format, long-acting PrEP remains largely unacceptable to transgender populations due to PrEP stigma, mistrust in medicine, and concerns about contraindications with gender-affirming hormone therapies (23–27). The objective of this study was to assess Black and Latina/e/x transfeminine adults’ preferences for a range of long-acting formats of PrEP, if they were to become available, in relation to short-acting PrEP, the daily oral pill.

## Materials and methods

### Survey setting, social positionality, and sample

Howard Brown Health (HBH) is a large federally qualified health center (FQHC) located in Chicago, Illinois. Established in 1974, a small group of white, cisgender, gay medical students aimed to respond more effectively and compassionately to increasing sexually transmitted infections among gay men. In its first 25 years, HBH became a critical node for LGBTQ+ health research in the U.S. through participation in Hepatitis B vaccine trials and, subsequently, in the Multicenter AIDS Cohort Study. Acquiring FQHC status in 2015, care at HBH is accessible to patients of all sexual orientations and gender identities and continues to prioritize LGBTQ+ populations. In addition to primary care, HBH offers gynecological care, walk-in STI/HIV testing, behavioral health care, gender-affirming care and surgical navigation, and dental care. From 2011 to 2021, the FQHC’s number of unique patients grew four-fold from approximately 6,800–30,000. In 2023, 37,382 unique patients visited one or more of its 11 clinics located across the city of Chicago, and 2,479 were identified as TW, not including non-binary or gender-non-conforming patients assigned to male at birth.

The study team largely consisted of four HBH research staff who are queer, trans, and non-binary, and racially, three are white and two are Latinx. The team consulted quarterly with a Community Advisory Board (CAB) created specifically for this study. The CAB consisted of six transfeminine and one transmasculine Black and/or Latina/x adults, all of whom worked at Black- or Brown-, queer- or trans-led community-based organizations (CBOs). CAB meetings took place at CBOs, typically lasted 2–3 h, and included lunch or dinner together, and members were compensated at the rate of $50 per hour.

Between March and December 2023, the study team used purposive sampling to enroll 198 transfeminine adults. The final version of the survey and the recruitment materials were translated into Spanish so that Latina/e transfeminine adults who were not fluent in English could participate. In consultation with the CAB, the study team implemented the following inclusion criteria: HIV status neutrality, a Black and/or Latina/x/e transfeminine gender identity, ages 18–35, and currently living, working, socializing, or receiving services in the city of Chicago.

### Data collection and measures

The study team employed multiple modes of recruitment: (1) in-person at community spaces located on the West and South Sides of Chicago; (2) via phone call, text message, or email to eligible HBH patients who had indicated in their electronic health record that they were willing to be contacted regarding participation in research; and (3) via passing flyers at HBH clinics and at community spaces. Upon the completion of a brief digital screening, staff contacted interested participants deemed eligible via email, phone, or in person to confirm their personhood and answer any questions. Subsequently, staff provided eligible participants with a unique link to the confidential survey housed in RedCap. Participants reviewed and provided digital informed consent before beginning, and upon completion, each received a $50 digital Visa gift card. Checks for survey completion and possible fraud via adversarial actors were conducted regularly by a study team member who looked for duplicate contact information that may have been missed during screening, completion times under 10 min, and contradictory response patterns. When potential fraud was identified, the study team analyzed indicators together to determine the validity of the survey.

Importantly, we collaborated with a University of Texas at Austin study team that launched a similar study, open to all Texan transgender and non-binary adults, and launched 1 year prior to ours. With their consent and collaboration, we used their survey instrument as a foundation and tailored it to this study’s objectives, sample population, and context (24). The survey was further revised based on two rounds of pilot-testing by CAB members and HBH’s Latinx Affinity Group. Both the CAB and the affinity group assessed the survey for legibility, culturally a0ppropriate and accessible language, and potentially psychologically harmful questions. For example, our CAB advised against using the standard Adverse Childhood Experiences Scale (ACES) due to potentially harmful and illegible language; the CAB found the Centers for Disease Control and Prevention (CDC) Behavioral Risk Factor Surveillance System (BRFSS) ACE module to be more acceptable and appropriate for the sample population.

Besides PrEP preferences, survey domains assessed sociodemographics, HIV-related sexual and behavioral health, mental health, social determinants of health, access to gender-affirming care, experiences with discrimination, trauma, and violence. To measure depression and anxiety, we included the Patient Health Questionnaire-9 item (PHQ-9) (28), omitting the suicidality question, and the Generalized Anxiety Disorder 7-item (GAD-7) (29). The Alcohol Use Disorders Identification Test (AUDIT) and the Drug Abuse Screening Test-10 (DAST-10) were used to measure substance misuse (30, 31), and the following scales were used to measure experiences with trauma, discrimination, and social support: Everyday Discrimination (32), BRFSS Adverse Childhood Experience module (33), the Sexual and Gender Minority (SGM) Adverse Childhood Events (34), the Multidimensional Scale of Perceived Social Support (35), and a modified version of the Medical Mistrust Multiformat Scale (36). Similar to Schnarrs et al., we used six items and replaced the term “medical authorities” with “medical professionals” (24).

#### Measures of PrEP attitudes and behaviors

To assess attitudes toward PrEP as an HIV prevention practice, we used the PrEP Stigma and Positive Attitudes Scale, consisting of two subscales (12). One subscale consists of seven questions that measure stigma held toward PrEP use, and the other subscale consists of three questions measuring positive beliefs held around PrEP use. To measure behaviors around the daily oral PrEP pill, we asked if participants had taken PrEP in the last 30 days and, if so, how many doses had they missed in the last 7 days, how they obtained PrEP, and if they experienced any side effects. Those not currently taking PrEP were asked if they had ever taken PrEP, and what their reasons were for not taking PrEP.

#### Primary outcome: preferred PrEP format

We measured respondents’ PrEP preferences in two ways. First, we used 5-point Likert scale (i.e., very likely, somewhat likely, neutral, somewhat unlikely, and very unlikely) to measure the likelihood of taking each type of long-acting PrEP as well as the daily oral PrEP. Each format was briefly described in terms of dosage frequency and modality. For example, “The PrEP intermuscular (IM) injection is administered in a clinic with a needle/syringe injected in the buttock every TWO MONTHS.” These statements were prefaced with the following guidance: “In this section we would like you to consider your interest in trying different types of long-acting PrEP that do not need to be taken as often or in the same way as the daily oral pill. Most of these types of long-acting PrEP are not yet available. In these examples, please assume each type would be available at low cost or no cost and have similar effectiveness against HIV and similar possible side effects as the current PrEP, the daily oral pill.” After completing the Likert scale, participants ranked their top three preferred PrEP formats as well as their least preferred PrEP format. Participants then selected reasons for each ranking and were also provided with a text box to explain each choice in their own words.

#### Secondary outcomes: PrEP eligibility/PrEP is recommended, and currently taking PrEP

Although HBH clinical protocol calls for prescribing PrEP to any HIV-negative, adult patient who requests PrEP, we measured PrEP eligibility based on the 2021 Centers for Disease Control and Prevention (CDC) PrEP Clinical Practice Guidelines (37). Participants were queried for sexual behaviors—receptive anal or vaginal sex—with cisgender male partner(s) in the past 6 months and the questions included (1) HIV status of partner(s)—negative, unknown, or positive—and, if positive, whether or not they are virally suppressed; (2) whether or not condoms were used consistently, and if they have ever had condomless anal or vaginal sex; and (3) if one had been diagnosed with and treated for an STI in the context of those recent sexual partnerships. In addition, we included questions regarding the following: (1) engagement in sex work ever, (2) engagement in sex work in the last 3 months, (3) substance use before/during sexual encounters, and (4) two questions about sexual partners expressing transphobic emotional or verbal abuse related to their bodies or gender identities during sexual encounters. These additional questions assess HIV risks that have been established as unique to transgender populations (15, 38). Questions regarding sharing needles as well as accessing post-exposure HIV prophylaxis (PEP) were also asked.

Finally, we conducted a secondary analysis to identify associations with individuals who reported the current use of PrEP. This analysis may provide additional insights into their preferences or dislikes for long-acting formats of PrEP.

### Statistical analysis

As a status-neutral survey, we conducted statistical tests to compare distributions of sociodemographic characteristics between those who reported an HIV-negative status and those who reported living with HIV. Then, we excluded those living with HIV and assessed the HIV-negative group’s PrEP-related knowledge, attitudes, and behaviors, while comparing those who would be eligible for PrEP to those ineligible, according to self-reported responses to questions derived from CDC guidelines, plus transgender-specific HIV risk guidance (15, 38). We conducted bivariate and multiple logistic regression analyses to identify associations with the most preferred long-acting PrEP format. Finally, to evaluate the performance of CDC’s indications for identifying current users of PrEP, sensitivity (i.e., proportion on PrEP with any indication for PrEP), specificity (i.e., the proportion not on PrEP without any indication for PrEP), positive predictive value (PPV) [i.e., the probability that those on PrEP also reported PrEP indication(s)], and area under receiver operating characteristic (ROC) (i.e., a measure of overall performance for predicting who will be on PrEP) were calculated (15). Analyses were conducted in SAS Software, Version 9.4. The study was approved and deemed exempt by the Howard Brown Health Institutional Review Board (Study #23-238).

## Results

Of 198 participants, 20.2% (*n* = 40) reported living with HIV (see Table 1). Compared to the HIV-positive respondents, those reporting an HIV-negative status were younger, had more formal education, were more likely to report a Bi+ sexual orientation, and had a racial/ethnic distribution higher in Latina/x and multiracial participants. Of the 52 Latina/x/e respondents, four took the survey in Spanish; one of the four was living with HIV, three others had tested negative within 6 months of taking the survey, and one reported currently taking PrEP. There were no significant differences (*p* < 0.05) between mean scores of HIV-positive and negative respondents for depression [7.5 (SD:7) vs. 6.3 (SD:7)], anxiety [7.1 (SD:6) vs. 6.1 (SD:6)], experiences with everyday discrimination [15.5 (SD:12) vs. 14.7 (SD:12)], medical mistrust [18.5 (SD:5) vs. 17.2 (SD:6)], and SGM Adverse Childhood Events [13.5 (SD:10) vs. 13.4 (SD:10)].

**Table 1:** Sample sociodemographic characteristics of transfeminine respondents to an online survey conducted in Chicago in 2023 regarding knowledge and experience with HIV pre-exposure prophylaxis medication—the daily pill—as well as preferences for longer-acting formats.

Sample characteristic	Total		HIV status				Test statistic	
			HIV negative		Living with HIV		t-test	*p*-value
	*N*	%	*n*	%	*n*	%		
	198	100.0	158	79.8	40	20.2		
Mean age in years (SD)	27.5 (4.6)		27.2 (4.6)		29.0 (4.1)		−2.27	*0.02*
Age groups							chi-sq	*p*-value
18–24	48	24.2	43	27.2	5	12.5	4.28	0.12
25–29	76	38.4	59	37.3	17	42.5		
30–35	74	37.4	56	35.4	18	45.0		
Race/Ethnicity								
Black	118	59.6	85	53.8	33	82.5	13.44	*0.004*
Latina/x/e	52	26.3	48	30.4	4	10.0		
Multi-racial	25	12.6	23	14.6	2	5.0		
Unknown	3	1.5	2	1.3	1	2.5		
Sexual orientation								
Asexual	4	2.0	2	1.3	2	5.0	10.84	0.09
Bi+	90	45.5	76	48.1	14	35.0		
Gay	31	15.7	22	13.9	9	22.5		
Straight	44	22.2	33	20.9	11	27.5		
Lesbian	11	5.6	11	7.0	0	0.0		
Same-gender loving	8	4.0	7	4.4	1	2.5		
Questioning or unknown	10	5.1	7	4.4	3	7.5		
Education								
H.S. graduate or less	75	37.9	55	34.8	20	50.0	10.79	*0.005*
Some college	80	40.4	62	39.2	18	45.0		
Bachelors or higher	43	21.7	41	26.0	2	5.0		
Housing								
Stable housing	119	60.1	93	58.9	26	65.0	0.51	0.48
Unstable housing	79	39.9	65	41.1	14	35.0		
Employment								
Full-time	68	34.3	58	36.7	10	25.0	2.02	0.16
Part-time or none	130	65.7	100	63.3	30	75.0		

Italicized values reflect statistical significance at *p* < 0.05.

Among those reporting an HIV-negative status (*n* = 158), 43 (27.2%) reported currently using PrEP. Although a range of reasons were given for not using PrEP, most pertaining to the perception that the type of sexual partners engaged in the last 6 months or the number of them were inherently low risk (e.g., partner is virally suppressed, in a monogamous relationship, and not currently sexually active).

Although 43 respondents were currently taking PrEP, 76 (48.1%) of the HIV-negative respondents met the CDC’s criteria for a recommendation to take PrEP (see Table 2) (39). Of the 43 respondents currently taking PrEP, 23 (53.5%) reported behaviors that met the CDC criteria for PrEP. Currently, taking PrEP is not associated with meeting CDC criteria for PrEP. The three survey items querying for HIV risk behaviors unique to transgender adults (i.e., not part of the 2021 CDC guidelines)—sex work ever with a cis male, sex work with a cis male in past 3 months, and having experienced transphobic abuse with a sexual partner—were each positively associated with meeting CDC’s criteria for a PrEP recommendation. A PrEP stigma score > 3 was the only negative association with meeting criteria for CDC PrEP recommendation [OR = 0.28, 95% CI (0.09–0.92)].

**Table 2:** Self-reported PrEP-related knowledge, attitudes, and behaviors across two Black and Latina transfeminine subgroups: PrEP-recommended and PrEP-not-recommended per 2021 CDC clinical guidelines for PrEP.

PrEP knowledge, attitude, behavior	Total		CDC recommends PrEP				Odds ratio & 95% CI			Test statistic	
	HIV negative		Yes		No		OR	CI low	CI high	chi-sq	*p*-value
	*N*	%	*n*	%	*n*	%					
	158	100.0	81	0.5	77	0.5	–	–	–		
PrEP stigma > 3											
Yes	16	10.1	4	4.9	12	15.6	0.28	0.09	0.92	4.45	*0.035*
No	142	89.9	77	95.1	65	84.4	REF	–	–		
PrEP poz attitd > 3											
Yes	125	79.1	64	79.0	61	79.2	0.987	0.458	2.127	0.00	0.974
No	33	20.9	17	21.0	16	20.8	REF	–	–		
HIV test (6 mo.)											
Yes	123	77.9	71	87.7	52	67.5	3.41	1.51	7.72	8.70	*0.003*
No	35	22.2	10	12.4	25	32.5	REF	–	–		
Heard of PrEP											
Yes	134	84.8	67	82.7	67	87.0	0.71	0.30	1.72	0.56	0.453
No	24	15.2	14	17.3	10	13.0	REF	–	–		
On PrEP (30 days)											
Yes	43	27.2	23	30.3	20	24.4	1.46	0.72	2.97	1.11	0.292
No	115	72.8	53	69.7	62	75.6	REF	–	–		
Sex work ever											
Yes	51	32.3	35	43.2	16	20.8	2.90	1.43	5.87	8.77	*0.003*
No	107	67.7	46	56.8	61	79.2	REF	–	–		
Sex work (3 mo.)											
Yes	17	10.8	14	17.3	3	3.9	5.15	1.42	18.72	6.21	*0.013*
No	141	89.2	67	82.7	74	96.1	REF	–	–		
Transphobic abuse											
Yes	56	35.4	35	43.2	21	27.3	2.03	1.04	3.95	4.32	*0.038*
No	102	64.6	46	56.8	56	72.7	REF	–	–		

Italicized values reflect statistical significance at *p* < 0.05.

**Figure 1:**
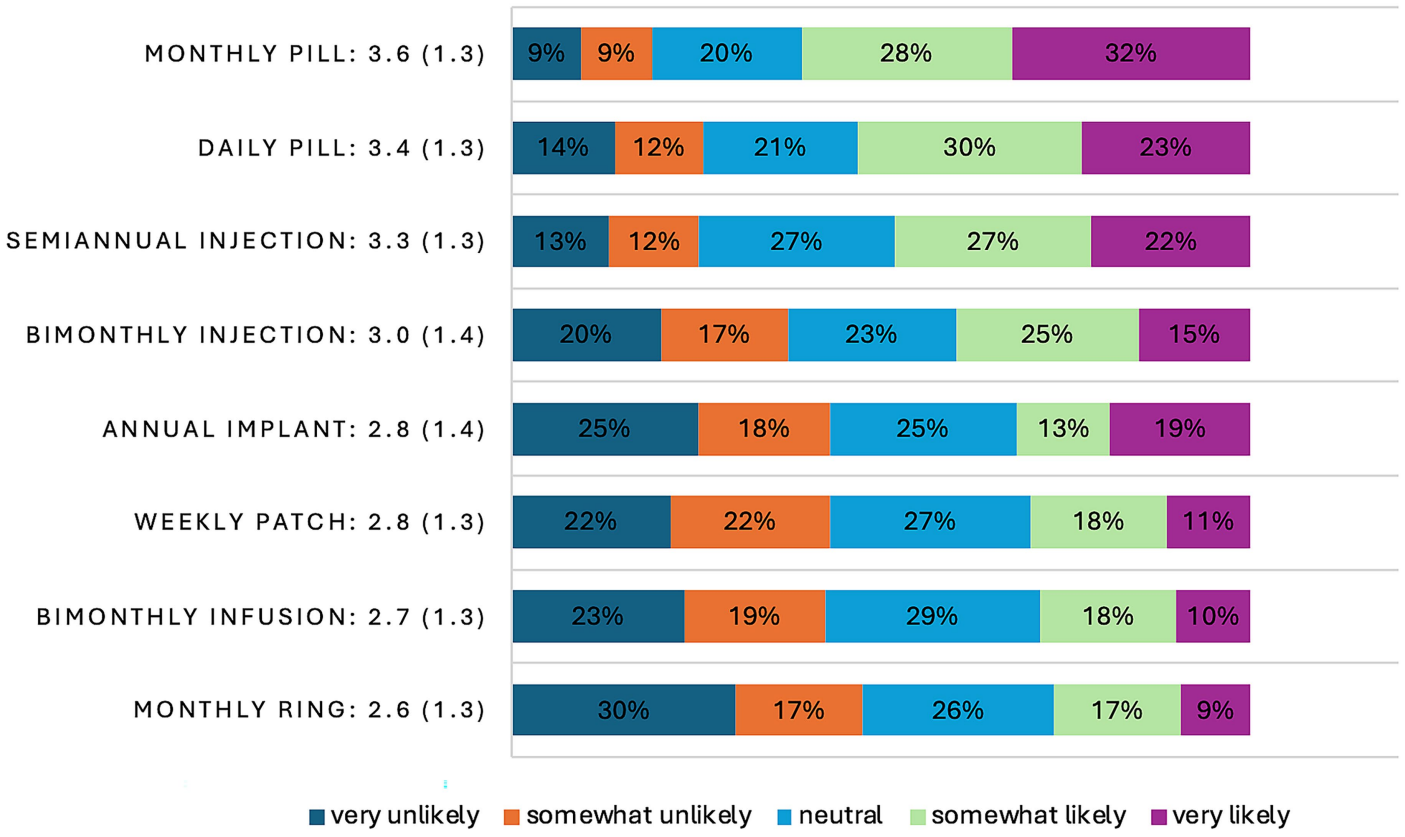
Bar graph of Likert score distribution on a scale of 1–5 of HIV-negative, Black, and Latina/x/e transfeminine survey participants (*N* = 158) that reflect their likelihood for taking a hypothetical long-acting format of PrEP as well as the existing short-acting format of PrEP, the daily pill in 2023. Included with each PrEP format descriptor is the mean score, along with its standard deviation in parentheses.

One form of long-acting PrEP—the monthly oral pill—was preferable to the daily pill (see Figure 1). The top three choices based on mean Likert scores—the monthly pill (3.6), the daily pill (3.4), and the semi-annual injection (3.3), respectively—exceeded a mean and a median score of neutrality, or 3.0, but none exceeded 4.0. HIV-negative respondents’ mean Likert scores on all other long-acting formats skewed toward unlikely uptake if they were to become available. For the top choice, the monthly pill, 96 (60.8%) reported that they would be very likely or somewhat likely to take it if available. The ranked choices reflected similar results, with the monthly pill being the top choice (24.1%) and the top-performing second choice (20.9%), while the patch was the top-performing third choice (24.1%). Reasons for favoring the monthly pill over other formats included anticipated improved adherence, ease of use, less invasive, less painful, and more privacy afforded. As one participant noted in an open text field regarding the rationale for their top choice, the monthly pill offers “long lasting protection with the least amount of medical intervention.”

Invasiveness, pain, and inconvenience were the primary reasons selected and articulated for the least favored format of PrEP in both sets of rankings—the monthly, event-based ring. The notion of having to insert and extract the ring from one’s anal or vaginal cavity around sexual encounters was described as messy, inconvenient, and unreliable in the open text field. Similarly, having minor but potentially painful and invasive surgery, sitting in a clinic for hours for a bi-monthly transfusion, or wearing a potentially uncomfortable and noticeable patch were also articulated as reasons for ranking long-acting formats last. Many respondents also said that they do not like the pain of needles and intramuscular injections.

In bivariate logistic regression analyses (*n* = 158), both higher education and full-time employment were associated with reporting a likelihood of taking the monthly pill if available (see Table 3). Controlling for age group, race, and sexual orientation, having a college degree (aOR = 6.73 [95% CI: 2.18–20.81], *p* < 0.001) and full-time employment (aOR = 2.70 [95% CI: 1.19–6.17], *p* = 0.018) were associated with expressing a likelihood of taking the monthly pill if it were available.

**Table 3:** Frequency distributions and bivariate analysis of independent variables modeled as predictors of HIV-negative, Black, and Latina transfeminine survey participants’ (*N* = 158) reported likelihood to take long-acting PrEP in the form of a monthly oral pill and associated odds ratios (ORs) and 95% confidence intervals.

HIV-negative	Total		Monthly PrEP Pill					95% CI		
Sample characteristic			Interested		Not interested		OR	Lower	Upper	*p*-value
	*N*	%	*n*	%	*n*	%				
	158	100	*96*	*60.8*	*62*	*39.2*	*–*	*–*	*–*	*–*
Age groups										
18–24	43	27.2	22	22.9	21	33.9	REF	–	–	0.3231
25–29	59	37.3	38	39.6	21	33.9	1.727	0.775	3.848	
30–35	56	35.4	36	37.5	20	32.3	1.718	0.764	3.863	
Race/Ethnicity										
Black	85	53.8	48	50.0	37	59.7	0.998	0.394	2.527	0.4034
Latina/x/e	48	30.4	34	35.4	14	22.6	1.868	0.665	5.247	
Multi-racial	23	14.6	13	13.5	10	16.1	REF	–	–	
Unknown	2	1.3	1	1.0	1	1.6	0.769	0.043	13.866	
Bisexual +										
Yes	76	48.1	51	53.1	25	40.32	REF	–	–	0.117
No	82	51.9	45	46.9	37	59.68	1.677	0.879	3.202	
Education										
H.S. graduate or less	55	34.8	24	25.0	31	50.0	REF	–	–	*0.0003*
Some college	62	39.2	36	37.5	26	41.9	1.788	0.859	3.725	
Bachelors or higher	41	26.0	36	37.5	5	8.1	9.300	3.169	27.291	
Housing										
Stable housing	93	58.9	60	62.5	33	53.2	REF	–	–	0.2482
Unstable housing	65	41.1	36	37.5	29	46.8	0.683	0.357	1.305	
Employment										
Full-time	58.0	36.7	46	47.9	12	19.4	3.833	1.817	8.087	*0.0004*
Part-time or none	100.0	63.3	50	52.1	50	80.7	REF			
Tested for HIV (6 mo.)										
Yes	123	77.9	73	76.0	50	80.7	REF	–	–	0.497
No	35	22.2	23	24.0	12	19.4	0.762	0.347	1.670	
PrEP taken (30 days)										
Yes	43	27.2	29	30.2	14	22.6	REF	–	–	0.2943
No	115	72.8	67	69.8	48	77.4	1.484	0.710	3.103	
Medical mistrust										
Yes	59	37.3	36	37.5	23	37.1	REF	–	–	0.9592
No	99	62.7	60	62.5	39	62.9	1.017	0.526	1.969	
PrEP Rx recommended										
Yes	81	51.3	50	52.1	31	50.0	REF	–	–	0.7981
No	77	48.7	46	47.9	31	50.0	1.087	0.574	2.059	

Italicized values reflect statistical significance at *p* < 0.05.

Given the relative popularity of the daily pill compared to long-acting formats, we conducted an additional analysis of two HIV-negative subgroups—those currently taking PrEP (*n* = 43) and those who were not (*n* = 115). Sample characteristics did not differ overall, with one notable exception. First, those taking PrEP were disproportionately stably housed compared to those who were not currently taking PrEP (76.7% vs. 52.1%, *p* = 0.007). Nearly all those who were currently taking PrEP reported having had an HIV test in the last 6 months (90.7% vs. 73%, *p* = 0.024). However, there was no difference between groups in terms of being recommended for PrEP based on CDC guidance (currently on PrEP: 53.5% vs. not on PrEP: 46.1%, *p* = 0.408).

With similar levels of PrEP indicated based on CDC guidance, between those currently taking PrEP and those not taking PrEP, we calculated sensitivity, specificity, the PPV, and the AUROC. The CDC guidance had fair sensitivity (0.5349, 95%CI: 0.3858–0.684) and specificity (0.5391, 95%CI: 0.448–0.6302). In other words, 53% of participants taking PrEP had at least one indication for PrEP, while 54% of participants not taking PrEP had no indications for PrEP (see Table 3). The PPV (0.30) and AUROC (0.6256) also reflect the fair performance of CDC guidance in identifying respondents who were taking daily PrEP.

## Discussion

Our study findings suggest that Black and Latina/x/e transfeminine adults’ current use of the PrEP daily pill, as well as their preferences for a long-acting PrEP monthly pill format over the daily pill, may be associated with social determinants of health (SDoH). Our sample had relatively high levels of full-time employment (37%) and higher education (65%), and these two social determinants may be key drivers of HIV prevention among Black and Latina/e/x transfeminine adults. A college degree (26%) was associated with HIV-negative status. Among HIV-negative respondents, a college degree, full-time employment, and having ever been tested were associated with reporting a likelihood of taking the monthly pill if it were to become available. In the U.S., full-time employment is the primary pathway to private healthcare insurance and access to care. Additionally, having stable housing was associated with the current uptake of the daily pill. However, the low prevalence of current PrEP uptake (27%) and potential uptake (60%) of a monthly PrEP pill is also consistent with the high prevalence of HIV (20%) in this Chicago sample and in the population of younger Black and Latina/x transfeminine adults across the United States. The relatively low mean Likert scores for these two most favored forms of PrEP—3.6 for the monthly pill and 3.4 for the daily pill—further highlight a possible need for additional HIV prevention resources.

These findings align with similar studies with transfeminine populations (15, 26) as well as ones (10, 25, 40) regarding other priority populations, where education, employment, and stable housing have been associated with PrEP uptake or preferences for long-acting formats over the daily pill. Structural factors appear to limit the reach and impact of efficacious biomedical and behavioral interventions, such as PrEP. The societal barriers to HIV prevention may explain why the 2021 CDC clinical guidance for effective PrEP prescribing did not perform much better than its chance at predicting who in our sample reported currently taking PrEP. At the same time, self-report does not constitute a clinical biomarker for identifying HIV vulnerability; additionally, providers such as Howard Brown Health provide PrEP to any HIV-negative patient who requests it. This harm reduction approach to HIV prevention provides critical reach, but more resources may be needed to reach those participants who were not currently taking PrEP but reported behaviors indicated for PrEP.

Overall, our sample had high mean scores related to trauma, discrimination, and mistrust in medical professionals and institutions. Such scores have been robust predictors of low utilization of healthcare and, combined with socioeconomic barriers to care, may help explain the low uptake of daily PrEP and the low likelihood of the future use of long-acting PrEP formats (12, 41). While we might liken our findings to a SDoH syndemics, other studies have identified a psychosocial and behavioral syndemics as a driver of HIV among TW: factors such as substance misuse, depression, childhood sexual abuse, and interpersonal violence have been linked to their HIV risk behaviors (42–44). The social facilitators of HIV prevention that we identified—employment, education, and stable housing—afford greater access to care and perhaps a greater understanding of PrEP as a prevention mechanism, as well as increased privacy and stability for taking and adhering to PrEP medication.

Community-engaged PrEP implementation strategy development may offer the most innovative and effective path forward. At a roundtable convened to discuss preliminary findings, community members expressed a dislike of condoms but also their preference for condoms over PrEP. PrEP requires a higher level of engagement with medical providers, clinical settings, and transit options perceived as potentially dangerous or threatening. In addition to community feedback, our CAB’s input was also a critical study element that helped build community, health literacy, and social support into this study and the participants. In particular, the shift to status-neutral eligibility criteria eliminated the study’s role in perpetuating the stigma of living with HIV through exclusion from our study. The same could be said for the shift from *transgender women* to a broader *transfeminine* gender identity eligibility criterion. In the future, studies like these, and especially implementation science studies, should consider ways to enhance the participation of Black and Latina transfeminine individuals who are living with HIV and their contributions of knowledge and lived experience.

Ideally, Black and Latina transfeminine people can play a larger role in the entire research process, not just as survey participants or CAB members. More robust university-community partnerships to build research capacity within communities are critical to implementation science and health equity. For example, one CAB member is currently an inaugural community research fellow at a local university’s school of medicine. Both funders and research institutions have begun to prioritize community-engaged research as well as its complexity in relation to executing reliable and valid process and outcome measures of community engagement. To this end, a compelling conceptual framework to guide the identification and specification of mechanisms between structural drivers and lived experience at marginalized intersections will help overcome these challenges with measurement and research equity (45).

Our study is not without limitations. Sampling was purposive, and the survey was cross-sectional and self-administered; this study design limits generalizability, and causality cannot be inferred. In addition, the survey was conducted during a time when the research team’s institution was experiencing highly publicized labor issues. These issues impacted staff’s internal relations as well as external relations with transgender communities and transgender communities of color, in particular. A measure of mistrust in research may help identify the impacts of any mistrust generated by research contexts, teams, or institutions. In addition, when the survey launched, only one of the long-acting formats of PrEP was available—the bimonthly injection—and, therefore, the other options were, and largely remain, hypothetical medications that have to be imagined as real. It is somewhat not surprising then that the most preferred long-acting formats were the most cognitively accessible ones; the monthly oral pill and the semi-annual injectable were the only formats that competed with the daily pill. The status-neutral inclusion criterion required excluding those living with HIV from the sections regarding PrEP and reduced our statistical power to detect differences and effect sizes in the sample. As a late modification, it also prevented us from modifying the survey to leverage more fully the knowledge and insights of those participants living with HIV.

## Conclusion

With current waves of anti-transgender and anti-diversity policies sweeping across the United States, social determinants of HIV prevention such as access to care, housing, employment, as well as education may become even more inaccessible. Transgender populations, most vulnerable to HIV, discrimination, and violence, will need more than biomedical interventions, such as short- and long-acting PrEP, to end HIV incidence. Innovative interventions and community-engaged implementation science research are needed to ensure access to life-preserving biomedicine as well as the community and social supports that will enhance their health and wellbeing.

## Data Availability

The raw, de-identified data supporting the conclusions of this article will be made available by the authors upon request.
